# The psychological burden of nutritional treatment in inherited metabolic disorders: Navigating daily life

**DOI:** 10.1016/j.ymgmr.2026.101365

**Published:** 2026-09-25

**Authors:** Annieke Venema, Frederiec K. Withaar, Foekje de Boer, Rianne M. Cnossen- Bosma, Terry G.J. Derks, Danique Draaisma- van Vliet, Marieke J. Fokkert-Wilts, Yteke Hoekstra, Melanie M. van der Klauw, Charlotte M.A. Lubout, Francjan J. van Spronsen, Andrea B. Haijer-Schreuder

**Affiliations:** a Department of Metabolic Diseases, Beatrix Children's Hospital, University Medical Center Groningen, University of Groningen, Groningen, the Netherlands; b UMCG Center of Expertise for Carbohydrate, Fatty Acid Oxidation and Ketone Bodies Disorders, University Medical Center Groningen, Groningen, the Netherlands; c UMCG Center of Expertise for Defects in Amino Acid and Organic Acid Metabolism, University Medical Center Groningen, Groningen, the Netherlands; d Department of Endocrinology and Metabolic Diseases, University of Groningen, University Medical Center Groningen, Groningen, the Netherlands; e Department of Medical Psychology, University Medical Center Groningen, University of Groningen, Groningen, the Netherlands

**Keywords:** Inherited metabolic disorders, Nutritional treatment, Qualitative research, Semi-structured interviews, Eating problems, Social restrictions, Quality of life, Burden

## Abstract

**Introduction:**

Inherited metabolic disorders (IMD) are a heterogeneous group of rare diseases, many of which require medically prescribed nutritional treatment. Maintaining such treatment can impose a psychological burden on people with IMD and their caregivers. Previous research in our center highlighted the need for deeper insight into this burden.

**Method:**

This study consisted of a scoping review and semi-structured interviews. A scoping review, conducted in PubMed and PsycINFO, focused on psychological outcomes related to nutritional treatment. Semi-structured interviews were held with people with IMD receiving nutritional treatment and their caregivers, exploring experiences of living with and managing nutritional treatment. Interviews were analyzed qualitatively following Braun & Clarke's principles.

**Results:**

The scoping review initially identified 2562 records, of which 52 articles were included. Seventeen interviews were conducted, involving twenty participants aged 8 months to 51 years, representing diverse cultural and socio-economic backgrounds. The qualitative analysis resulted in five main themes.

**Conclusion:**

Nutritional treatment in IMDs is often experienced as burdensome and can affect psychological well-being. Participants reported daily inconveniences, social limitations, and reduced spontaneity. At the same time, many described eventually finding ways to manage the treatment and reaching a level of acceptance, integrating both the IMD and its nutritional demands into their lives.

**Take-home message:**

Nutritional treatment in IMDs can place a substantial psychological burden on individuals and caregivers, yet many ultimately find ways to integrate these demands into daily life. Understanding this lived experience is essential to improving long-term support.

## 1 Introduction

Inherited metabolic disorders (IMD; ORPHA:68367) comprise a heterogeneous group of rare diseases and can be categorized into three main groups, namely intoxication type (e.g. phenylketonuria and organic acidurias), energy metabolism (e.g. hepatic glycogen storage disease (GSD) and fatty acid oxidation disorders) and complex molecules. Many of the IMDs of the first two groups can be treated with a lifelong medically prescribed nutritional treatment, which can consist of food restrictions, increased frequency of nutritional intake (during day and nighttime), dietary supplements and/or dietary products (e.g. low-protein bread) [1].

The primary goal of dietary and nutritional treatments is to prevent acute and chronic complications and improve clinical outcomes [1]. Despite strict adherence to treatment, people can still develop serious complications. For example, people with hepatic GSD can develop hypoglycemia and hepatic tumors [2], while people with phenylketonuria do not always note a deviation in their treatment at short notice and organic acidurias can develop multisystem diseases [3]. Maintaining strict nutritional treatment may impose a heavy psychological burden on patients and their family members and may impose stress on caregivers being responsible for raising a child with such IMD [4].

Several studies have reported lower health related quality of life (HrQoL) in people with IMD and their caregivers [5], [6]. The general QoL of children with IMD (children with PKU not included) treated with restrictive nutritional treatment was mostly impacted by anxiety and behavioral difficulties, high parental trait anxiety, and a younger age at diagnosis. In this study, psychological factors were found to be more important for determinants of QoL than clinical variables [7]. This is in line with a study on PKU. It was found that emotional and social outcomes tended to be poorer in adults with PKU who had lower blood Phe concentrations during childhood. It could be hypothesized that strictly and rigidly maintaining the nutritional treatment in infancy had its impact on stress and emotion regulation later in life [8]. Very recently, Sherlock et al. [4] conducted a systematic review on the psychosocial challenges faced by young people with IMD. It was concluded that this group experienced a diverse array of physical, social, psychological, and practical challenges, including social isolation, burden for caregivers, learning, and emotional difficulties.

Previous research by our center concluded that people with GSD experienced poor appetite, selective eating, slow eating and aversive symptoms during mealtimes, and several developed Avoidant/Restrictive Food Intake Disorder (ARFID) that required intensive clinical treatment. Additionally, people experienced sleeping problems and caregivers reported stress about their child's illness [9]. In a study conducted at our center, caregivers reported that their children with PKU experienced eating problems and more negative emotions concerning food and eating. Additionally, restrictions in social activities were more common than in healthy children [10]. These studies highlighted the need for greater insight into the emotional consequences of nutritional treatment in people with IMD to provide guidance to families and to improve clinical outcomes and QoL.

Considering the possible consequences of the strict nutritional management in IMD on psychosocial development, we have to realize that eating and food are key factors in social and emotional development [11]. Infants develop basic trust and self-soothing behaviours through regulation of hunger and satiety. Toddlers learn through modelling eating choices, patterns and behaviours with participation in family meals and develop food preferences by experimenting with and refusing different foods. Eating together leads to a feeling of connection and has an important role in the facilitation of social binding. Thereby, at least theoretically, following a strict dietary treatment may give rise to eating problems, interfere with normal psychosocial and emotional development, and increases the risk to feel inferior and socially excluded and placing a heavy burden on parents and may interfere with the bond between parents and their child with IMD.

To further investigate the life-long impact of strict dietary treatment in IMD on social emotional development and the specific challenges during the different stages of childhood, we conducted a study, comprising a scoping review and qualitative analyses of semi-structured interviews. Our hypothesis was that nutritional management affects the parent-child relationship and causes psychological distress at every stage of life. Psychological distress may lead to emotional problems in both patients and their families.

## 2 Methods

This study integrated a scoping review and semi-structured interviews, The development of interview questions was guided by recurring themes identified in the literature, while intentionally maintaining a broad scope to address substantial gaps and underexplored areas in current knowledge.

### 2.1 Ethics

The Medical Ethical Review Committee of UMCG concluded that this study fell outside the scope of the Dutch Medical Research Involving Human Subjects Act. For the semi-structured interviews, the audio and transcriptions were protected and only available for the research team. Informed consent was obtained from patients and/or caregivers before the interviews, and the participants had the right to end their participation at any time.

### 2.2 Scoping review

A scoping review of the psychological outcomes of people with IMD with strict nutritional treatment—and their caregivers, or siblings—was conducted. The search strategy, developed with an information librarian, included PubMed (searched on 18 November 2024) and PsycINFO (16 December 2024). Rayyan (https://www.rayyan.ai/) was used to remove duplicates and manage screening. Two reviewers (AV and FKW) screened the included records independently. At each screening stage, the records were assessed for relevance to IMD, and psychosocial outcomes or burden. In the final stage, full-text screening was assessed for psychological outcomes specifically linked to nutritional treatment, so for example studies on general QoL in IMD were excluded. Exclusion criteria were: [1] absence of a prescribed nutritional treatment, [2] reviews, dissertations, or textbooks, and [3] non-English publications [4] no explicit link of psychological outcomes to nutritional treatment. Systematic reviews were excluded if their original articles about nutritional treatment were already included in our search or were not relevant to our research question. Discrepancies were resolved through discussion (with ABHS). The literature identified was used as background information to inform the development of the interview. Insights gained from this literature provided the basis for constructing the qualitative interview questions.

### 2.3 Semi-structured interviews

#### 2.3.1 Participants and procedure

Eligible participants for the study were people with IMD of all ages, with medically prescribed nutritional treatment and their caregivers. All people (children and adults) were treated at the Department of Metabolic Diseases, Beatrix Children's Hospital, UMCG.

Our specific starting point was a breakdown into age groups, and then, within each age group, a variety of IMDs and people from different cultural backgrounds. Based on those criteria, we began filling the matrix according to the chronology of scheduled outpatient visits or admissions to the ward over a 2-month period. After those 2 months, we filled any remaining slots in the matrix by specifically searching for that particular age group and that specific condition.

All eligible subjects and families were contacted by the metabolic nurse (RCB) to provide verbal and written information about the study and to invite patients and their caregivers to participate.

All interviews were conducted by AV, together with FKW or a medical social worker (MHV). AV had no clinician-patient relationship; engagement with participants occurred only within the research project. The interviews took place at the outpatient clinic or via Microsoft Teams. Support from an interpreter was available when needed. All the interviews were audio-recorded and transcribed verbatim by AV, FKW and ABHS.

### 2.4 Interviews

The semi-structured interview was developed by a team of metabolic pediatricians, dietitians, a medical social worker, a medical PhD student (AV), and a psychologist (FKW). The interview contained the following topics: practical organization of the nutritional treatment, challenges during illness episodes, restrictive impact of the nutritional treatment, treatment adherence and responsibility, quality of sleep, quality of life, emotional impact (mood, anxiety, stress), self-image and self-esteem, need for psychosocial professional help, subjective experiences at the (outpatient) clinic, and resilience, qualities and strengths. The interview format can be found as Supplementary file 1 (written in Dutch).

### 2.5 Data analysis

The transcripts were analyzed using thematic analysis as described by Braun and Clarke [12]. Independent, concurrent coding was performed using Atlas.ti (https://atlasti.com). During step 1 (familiarization), we already formulated our initial ideas for themes. For help with the iterative thematic analysis, we imported the initial codes (anonymized and without the quotes) to Gemini (https://gemini.google.com). We did not use AI in step 1 (familiarization) as our interviews were privacy sensitive. We prompted Gemini into the steps of Braun and Clark, aim of the study, and research questions and our own thoughts about themes. We gave instructions to further conceptualize the themes, focusing on previously unidentified themes and clustering of themes and topics. At every step, we checked how Gemini had clustered the codes into themes, and based on that, we entered increasingly targeted prompts so that Gemini could identify even more specific themes. We also critically assessed the extent to which an additional step involving overarching themes still led to meaningful additional themes, or whether it became so general that relevant information was lost. We went through all the codes multiple times during the process to make sure they could be categorized under the appropriate themes and that no information was lost.

### 2.6 Themes

were further discussed, refined and reformulated by the principal investigators and subsequently together with the multidisciplinary team consisting of metabolic pediatricians, dietitians, nurses, clinical (neuro)psychologist and social workers.

## 3 Results

### 3.1 Scoping review

The initial search yielded 2562 records; a total of 52 articles were eventually included for analysis. The selection process is illustrated in Fig. 1. One English written systematic review from Japan was included, as it covered articles originally written in Japanese [13]. In our scoping review, most studies were performed on PKU (*n* = 27) or IMD as an overall group (*n* = 12). The other publications described the following diseases: tyrosinemia type I, urea cycle disorders, familial chylomicronemia syndrome, galactosemia, hepatic GSD, idiopathic ketotic hypoglycemia, long chain fatty acid oxidation disorders, lysosomal acid lipase deficiency, lysosomal storage disorders and maple syrup urine disease (MSUD). Most studies used questionnaires or interviews (semi-structured, in-depth interviews or focus groups) to assess psychological outcomes and burden of nutritional treatment.

**Fig. 1:**
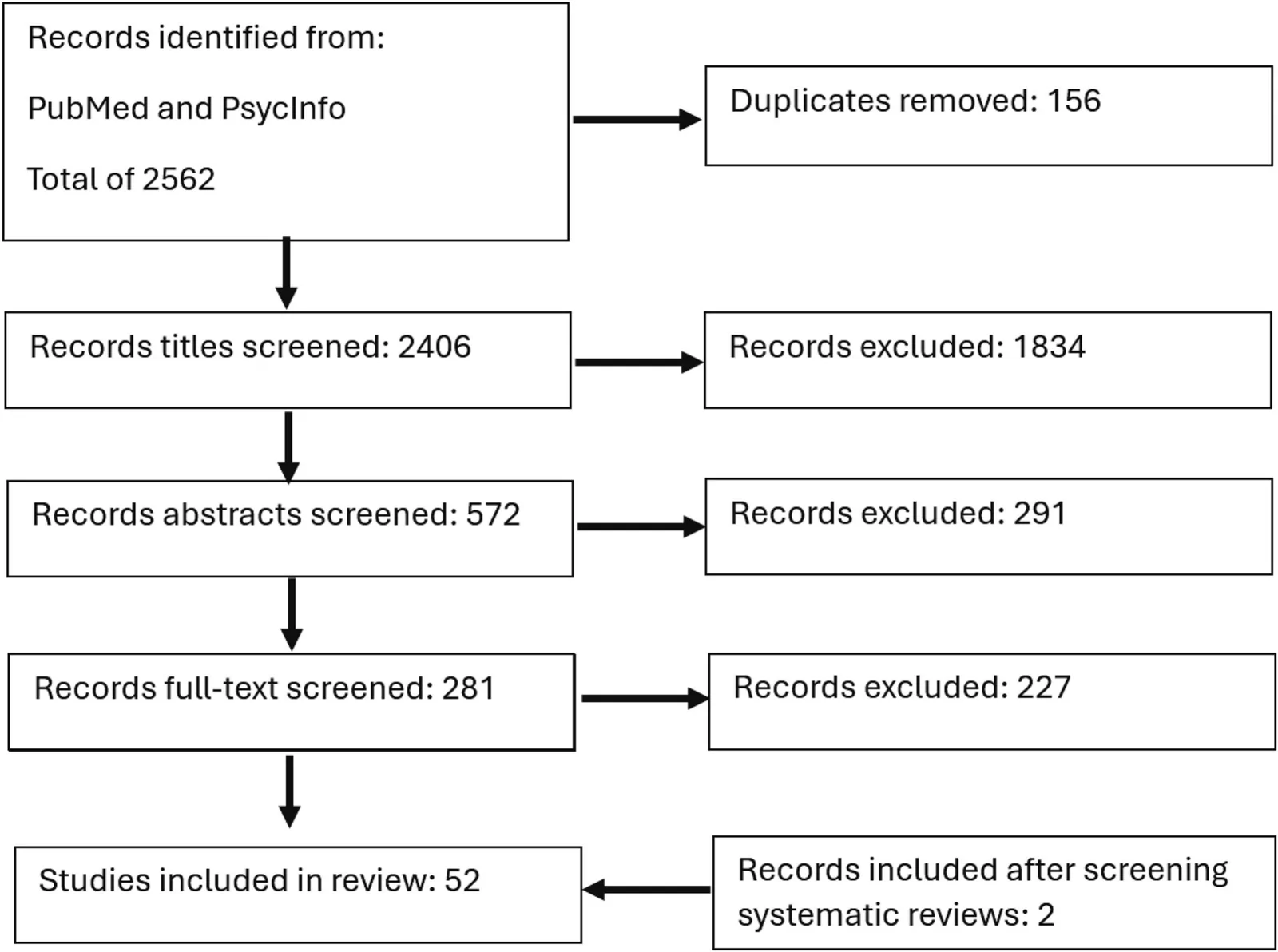
Flowchart of the record screening of the systematic review.

#### 3.1.1 PKU

In the 31 studies on PKU, general health-related quality of life (HRQoL) was largely unaffected by nutritional treatment [14], [15], [16], though strict dietary restrictions did reduce food enjoyment and posed practical challenges [17], [18], [19], [20], [21], [22]. People with PKU and caregivers reported stress around limited food variety, organizing meals outside the home, and managing supplemental formulas, with social events often complicated by dietary needs [10], [15], [20], [23], [24], [25]. Despite these burdens, most people accepted nutritional treatment as essential for cognitive health and academic success [26]. Caregivers—especially mothers—often struggled with relinquishing control, leading to feelings of guilt, anxiety, and social discomfort, while people sometimes felt different or excluded [17], [26], [27], [28], [29], [30], [31], [32], [33], [34], [35], [36], [37]. Nevertheless, most people achieved autonomy and reported high self-esteem, with no clear link between nutritional treatment and symptoms of depression or anxiety [18], [31], [38].

#### 3.1.2 IMD

In the literature where IMD was described as a group, it emerged that patients and their caregivers often experience reduced HRQoL due to the burden of disease and strict nutritional treatment [6], [39]. Studies report heightened anxiety, emotional distress, and social challenges, especially among caregivers managing infant nutritional treatment or emergency dietary needs [7], [13]. Nutritional treatment is frequently perceived as more burdensome than medical treatment, affecting daily routines, school integration, and social participation [13], [40], [41], [42], [43]. Common issues include poor appetite, limited food variety, long mealtimes, and feeding difficulties, particularly in tube-fed children [43], [44].

#### 3.1.3 Other IMD

Flanagan [45] found elevated dysfunctional attitudes toward food and lower body esteem in patients with GSD. Venema [9] even found a high prevalence of avoidant/restrictive food intake disorder (ARFID) or multiple ARFID characteristics such as poor appetite, selective and slow eating habits and aversive reactions during mealtimes in GSD patients. Nutritional treatment in specific disorders such as tyrosinemia, galactosemia, and MSUD are linked to guilt, frustration, and social exclusion [46], [47], [48], [49], while conditions like long-chain fatty acid oxidation disorders and familial chylomicronemia syndrome demand rigid meal planning and limit spontaneity [50], [51], [52]. Despite some variation—such as more manageable experiences in LAL-D or mild burden in urea cycle disorders—many families report emotional strain, disrupted family dynamics, and reduced personal time [53], [54]. Support from family and social networks plays a crucial role in coping with these challenges.

Supplementary file 2 provides a detailed summary of all included studies, outlining patient characteristics and key outcomes relevant to psychological burden of the nutritional treatment.

### 3.2 Semi-structured interviews

#### 3.2.1 Participants

Seventeen interviews were conducted, encompassing twenty participants ranging in age from 8 months to 51 years and varying in cultural and socio-economic backgrounds, representing a diverse spectrum of IMD – primarily PKU and hepatic GSD, but also including methylmalonic aciduria (MMA), propionic acidemia (PA), MSUD, galactosemia and medium-chain acyl-CoA dehydrogenase deficiency (MCADD). The interviews took place from January to April 2025. Table 1 provides an overview of the participants.

**Table 1:** Background information of the participants.

Variable		Number
Sex	Male	10
	Female	10
Current age	Mean	16 years
	Median	13 years
	Range	0–51 years
Diagnosis	Galactosemia (ORPHA: 79239)	1
	Glycogen storage disease 0b (ORPHA:137625)	1
	Glycogen storage disease Ia (ORPHA:79258)	4
	Glycogen storage disease Ib (ORPHA:79259)	3
	Glycogen storage disease IX (ORPHA: 79240 and 264,580)	2
	Medium-Chain Acyl-CoA Dehydrogenase Deficiency (ORPHA: 42)	1
	Methylmalonic acidemia (ORPHA:289504)	2
	Maple syrup urine disease (ORPHA:268145)	1
	Propionic acidemia (ORPHA: 35)	1
	Phenylketonuria (ORPHA:79254)	4
Interviewed	People with IMD	5
	People with IMD and caregiver(s)	3
	Caregiver(s)	12

### 3.3 Qualitative analysis

The initial coding yielded 1550 code assignments, which were clustered in 60–70 unique codes. Through iterative thematic analysis, five overarching topics emerged within a total of 11 themes. Supporting quotations from various participants are presented at the end of the results section to illustrate the data (Table 2).

**Table 2:** Supporting quotes for the thematic analysis.

**Themes**
**1. Living with a Diet: Navigating Everyday Life**
**1.1 Diet as a Structural Element**
Parent: “Yeah, I wouldn't know how it would be any different. (…) I didn't work five days a week, but now I do work five days a week, but more in the mornings. So, yes, I've changed that.” (about how a mother adjusted her work schedule to support their son's diet at school).
Parents: “Yes, and at a certain point that creates so much adaptability that in the beginning you just let those social things go, yes.”
Parent: “Yes, just at a restaurant, it depends a bit on where we're going… because we also like to call ahead. It goes like this: can we bring our own bread, or is it a problem if we bring our own chicken nuggets?”
**1.2 Social Integration versus Exclusion**
Parent: “We come from Turkish culture, right? And we have a much richer cuisine, and… It's not really a cheese sandwich. If only we'd stuck with that…”
Participant: “I'd sometimes tell my friends, “No, I'm not going out tonight.” Or “I'm not going out tonight because they were going to eat first and then go out.”
Parents: “Well, we don't have much of a social life. Since people found out the children were sick, yes, friends. Yeah… No, we don't really have any.”
**2. The Inner World: Psychological Impact and Emotional Acceptance**
**2.1 Self-Perception, Identity and Feeling Different**
Participant: “You really just want to be like everyone else. You also want to have something when there's a treat and not have them to buy you something sugar-free. That doesn't make you very happy. (…) You just want to be like everyone else and not stand out.”
Participant: “But I've always enjoyed the food I was allowed, so I never really had a problem with it. I wasn't jealous or anything? That people were eating cake.”
**2.2 Emotional Burden, Worries and Fear of Losing Control**
Participant: “Yes, and then there's that fear your parents have—and I think that's what affects you the most. They worry: Will you manage? Will you have everything you need? Will you eat on time and not do anything unusual? And when we're teenagers or pre-teens, sometimes we just want to push back or even lash out.” Parent: “She looks at the other nephews and nieces her age, and it's becoming very pitiful and difficult for her. Why can they eat and I can't? I find that truly heartbreaking.”
Parent: “The bond with mother and child is really damaged by warning him again and again, again… (…) Now he says, ‘I have my own opinion, I have my own way. Today I'm going to do it differently.’ Uh, what should I do? Yeah, you can't put him in jail for that.”
**2.3 Well-being and Regulation: Mood and Satisfaction**
Parent: “So you're always pushing her or something, well that goes a bit against your feelings, and you know you have to, so yeah that's just not very pleasant at all.”
Parent: “And that, uh, no, that can really make him angry sometimes. That's not allowed. Because he wants to share that with his sister. (…) Then it sometimes breaks within you, that you think, yeah…” (how a brother can't share his food with his sister)
**2.4 Disrupted Eating Patterns**
Participant: “As a child, I didn't eat much. I simply had a feeding tube. (…) And then, at twelve, I was already in high school, I thought, ‘It's time to start eating,’ yes, as a twelve-year-old who's hardly ever eaten. That was a huge change for me.”
Participant: “I don't know, I always find the term eating disorder a bit grandiose. I always call it disordered eating because I think I don't have to fit into a certain box, but there have definitely been times when I either vomited after a meal or something else.”
Parents: “Those unique flavors, sambal, lemon. (…) I think they have a certain, very strong aroma, and they like that, yes.”
**2.5 Disrupted Sleep Patterns**
Parent: “Sleep, yes, I found that really difficult. Especially when I had to go back to work (…) and I always had to get up in the night. So I had to set my alarm for one o'clock to give her a bottle, which meant she would sometimes stay awake for a long time, and my alarm would go off at 6:15 a.m., which was really tough.”
**3. Acceptance and Adaptation: Coping with IMD**
Participant: “Even though I ended up in the hospital, things didn't go well. (…) I've never had an issue with the fact that it happened there on vacation, so I don't avoid anything because of this previous situation.”
Parent: “I adapted my life to his situation, it's no different. I wanted it to be different, but this is the truth: I have a child, I've accepted this situation, and I'm getting help.”
Participant: “You have to be grateful for what you have, and that's how I raised him to be grateful for what you can do, what you have. And when I think about that, I'm happy again.”
**4. The External Network: Dependency and Support**
**4.1 Shared Responsibility and Social Network**
Parent: “We chose not to use daycare at all for the first two years. Because we were very worried that the food, well, they wouldn't pay enough attention.”
Participant: “I make sure I go (on holiday) with friends I really trust, but I make sure that they know me long enough, otherwise I would feel unsafe, because then I just know, what if something happens, then no one will take me into account”
**4.2 Communication and Contact with Healthcare Professionals**
Parents: “At one point, the nurse and doctor there also said, “Yeah, you know, you know more about this disease than we do. (…) I believe them right away. They were very honest about it, but yeah, the moment you see your child lying there so sick, you think, I'd rather have someone who knows more about this than I do.”
Parent: “She really likes it here, because the doctors do their best. (…) Everything I get from you regarding the diet is sufficient.”
**5. Stages of Life and Life Span**
Parent: “Well, more so when we're about to hit puberty, I'm a little scared of that. I do have that fear, thinking, “Oh, what's going to happen then? How will you handle that? How can I best support you through this?”
Parent: “Yeah, what will that do to a child socially? I've worried about that too, yes. (…) And what if he doesn't have any friends, and they ignore him, or if the parents find it too complicated? Yes, if he lets him play there or doesn't get invited to children's parties, he'll probably be left out. Yeah, there are those, yes. I did worry about that.”

**1. Living with Nutritional Treatment: Navigating Everyday Life**

This main theme highlights how nutritional treatment is integrated in daily life and the adjustments people must make.

**1.1 Nutritional treatment as a Structural Element**

A medically prescribed nutritional treatment needs extensive daily adjustments requiring planning, preparation and implementation throughout the day. One participant mentioned that nutritional treatment means “living by the clock”. Nutritional treatment requires adaptations in routines on school days, holidays or day trips, dining in a restaurant, and even in planning and organizing driving lessons. Examples given were calling a restaurant beforehand or adjusting working hours to support a child's nutritional treatment. Another respondent mentioned that the whole family had adapted their eating pattern to the child's nutritional treatment. Because extensive planning is required, spontaneous activities often cannot be undertaken. On the other hand, it was voiced that nutritional treatment also provides stability and structure in daily life.

**1.2 Social Integration versus Exclusion**

Food plays a central role in social life and in culture and therefore, medical nutritional treatment can interfere with participation and can lead to feelings of exclusion, particularly during sleepovers, celebrations, or even when eating together. Because the study included families from diverse cultural backgrounds, several Arabic respondents explained that their eating culture is richer than the Dutch one, adjusting the nutritional treatment more complex. However, small adjustments do help social inclusion and support shared experiences. One example given was offering a replacement treat at a birthday celebration at school. Some participants reported that nutritional treatment did not affect their social life, while others preferred to keep it private -particularly in peer or professional settings and in new social or romantic relationships.

**2. The Inner World: Psychological Impact and Emotional Acceptance**

This theme addresses the psychological and emotional aspects of living with nutritional treatment, like identity formation, emotional burden, and overall well-being.

**2.1 Self-Perception, Identity and Feeling Different**

Nutritional treatment can influence self-concept and create a sense of being different or standing out. For example, some caregivers needed to give extra attention to their child with IMD to maintain the nutritional treatment. In certain cases, feeling differently affected self-confidence and self-esteem. However, many participants did not perceive themselves as different from their peers.

**2.2 Emotional Burden, Worries and Fear of Losing Control**

The emotional cost of nutritional treatment can manifest as anxiety, worry, and fear of losing control. These emotional consequences may affect treatment adherence, and caregivers can struggle to relinquish control, often experiencing heightened vigilance. Some caregivers, for instance, co-slept with their children to monitor nutritional needs overnight. Others described the emotional difficulty of refusing certain foods to their child, which at times even affected the parent-child bond between the parent and the child. However, some participants reported no emotional difficulties related to nutritional treatment, for example because they understood the health consequences of not adhering to it.

**2.3 Well-being and Regulation: Mood and Satisfaction**

Despite the challenges, people with IMD often reported a high level of overall well-being. Although a life-long nutritional treatment was frequently described as undesirable, positive emotions generally predominated. Families noted that they had come to appreciate the small things in life and felt they functioned well as a team. Episodes of anger or frustration did occur but were usually short-lived. Several caregivers described mealtimes as less pleasant due to frustrations on both sides: children who were not allowed certain foods, and caregivers who felt pressured to ensure their child ate.

**2.4 Disrupted Eating Patterns**

Restrictions associated with nutritional treatment, such as the need for frequent meals, can disrupt normal eating patterns and may lead to reduced food variety, delayed development of autonomous eating behaviours, and altered taste preferences.

**2.5 Disrupted Sleep Patterns**

Participants noted that late-evening and nocturnal feeding requirements disrupted the sleep routines of both children and caregivers. In some cases, caregivers chose to sleep alongside their child to facilitate nighttime care and ensure adherence to the nutritional treatment and safety.

**3. Acceptance and Adaptation: Coping with IMD**

People frequently described a process of normalization and acceptance, often expressed as “It is what it is.” This reflects a strong capacity for psychological adjustment and resilience in coping with chronic nutritional treatment. Families demonstrated mutual care and support, highlighting the importance of shared experiences and peer support. Many respondents focused on the positive aspects of their situation and the development of strong interpersonal relationships.

**4. The External Network: Dependency and Support**

This theme addresses the role of the wider social network the reliance on external sources of support.

**4.1 Shared Responsibility and Social Network**

Sharing responsibilities for nutritional treatment with other caregivers – such as teachers, grandparents, and childcare staff - is important. At the same time, caregivers expressed concerns about doing so. Some had experienced difficulties and did not fully trust others to manage the nutritional treatment. Nonetheless, they acknowledged that a supportive network is essential for optimizing care.

**4.2 Communication and Contact with Healthcare Professionals**

Participants generally expressed satisfaction with the communication and continuity of care provided by specialized IMD healthcare teams. The dedicated focus on nutritional management was particularly valued and contributed to a sense of support. Challenges emerged during episodes of acute illness and hospital admissions in general hospitals, when caregivers sometimes felt they had more knowledge about nutritional treatment than the healthcare professionals.

**5.1 Stages of Life and Life Span**

This final theme highlights the evolving role of nutritional management across developmental stages, the anticipated future impact of the disease, and emerging challenges. Caregivers frequently expressed concerns about their children's long-term ability to maintain adherence to nutritional treatment, the financial burden of specialized products, and the gradual transfer of responsibility. Nonetheless, many reported that nutritional treatment became easier to manage over time, suggesting a generally positive long-term outlook.

All themes are presented in Table 2 and supported by illustrative quotations that reflect the depth and nuance of respondents' experiences.

## 4 Discussion

The aim of this study was to improve understanding of psychological outcomes, burden and experiences of people with IMD with a medically prescribed nutritional treatment.

Summarizing the findings from this mixed-methods study, nutritional treatment in IMDs can be described as burdensome and affecting psychological well-being. People describe daily inconveniences, social restrictions, and lack of spontaneity. Rigorous nutritional treatment planning and preparation continuously affect daily life, influencing routines such as (frequent) everyday meals, social outings, and holidays. When a child requires night feeding, this can lead to disrupted sleep patterns and chronic sleep deprivation. People with IMD often describe feeling differently from others and feeling socially excluded or uncomfortable when eating together with friends, family, or colleagues. Caregivers strive for a balance between social and emotional development, and adherence to nutritional treatment to maintain optimal metabolic control. People and caregivers may suffer feelings of guilt in case of non-adherence. Caregivers struggle with fear of losing control in handing over the responsibility of nutritional treatment to their child or adolescent with IMD. Periods of illness in which requirements of nutritional treatment cannot be met are especially distressing for caregivers due to fears of metabolic crisis or acute and chronic complications. At times people with IMD suffer from feelings of stress, anxiety, and frustration. It was reported that some people with IMD experienced a preoccupation with nutritional treatment, or even developed dysfunctional eating patterns.

On the other hand, people with IMD and their families report that, over time, they found their own way of managing the nutritional requirements. They reached a point of acceptance, viewing both the IMD and nutritional treatment as part of daily life and learning how to adapt. Families described relying on and supporting one another, often focusing on the positive aspects of life and expressing gratitude for being able to live a relatively normal life despite a serious illness. In the interviews, people with IMD and their caregivers demonstrated acceptance, habituation, and resilience. Some participants even reported that the nutritional treatment had no impact on their social life.

When comparing the outcomes of our scoping review and semi-structured interviews with previous research, clear similarities emerge. Carpenter [34] identified comparable themes in qualitative interviews with caregivers of children with PKU, including control, striving for normality, and acceptance of PKU. Shirdelzade [55] also reported overlapping themes, such as self-forgetting in the shadow of full-time care, passing difficulties in the duality of hope and hopelessness, and caring in a continuum of isolation –and socialization. However, several themes described in the Shirdelzade study were not reflected in our findings, including the notion of future tied to the past, disturbed psychological health in the shadow of a lost ideal child, and blaming as a way of escaping difficulties.

In our scoping review, most studies focused on individuals with PKU, possibly because PKU was the first treatable IMD, is relatively less rare and for several decades diagnosed by newborn screening in most countries worldwide. The apparent lack of impact of nutritional treatment on HRQoL reported in the PKU literature may be due to the use of questions that are insufficiently specific or concrete, as well as the fact that general HRQoL scores represent a composite of many factors, including those more specifically related to PKU. Another possible explanation is the relatively low incidence of acute complications in PKU compared with other IMDs or the fact that patients have become known to their issues. When PKU-specific questionnaires, interviews, or focus groups are used, however, the burden associated with nutritional treatment becomes clearly evident [10].

Several methodological limitations should be acknowledged. First, most participants had PKU or hepatic GSD, as the UMCG is an expertise center for these disorders. In addition, most participants were of Dutch origin. In the Netherlands food and nutritional moments are important but may be of less social importance if compared to other Mediterranean countries. This all may have influenced the interview findings. In some cases, children were interviewed together with their caregivers, which may have made children – particularly adolescents - less forthcoming. At the same time, this joint setting facilitated open dialogue between children and caregivers, yielding additional insights. Furthermore, the interviews did not allow for a clear distinction between burdens arising from the nutritional treatment itself and those related to the underlying disease. Metabolic disorders can affect the central nervous system and present with clinical features resembling psychiatric conditions, such as depression or anxiety. In addition, some IMDs – such as hepatic GSD and organic acidurias - may lead to acute complications including hypoglycemia and coma. These safety risks can also increase worry and psychological distress [2].

There are also limitations to using Gemini for the qualitative analysis of the interviews. There is a risk of reflexivity, which we have minimized as much as possible by reviewing all the codes ourselves multiple times and thoroughly checking the themes for completeness.

This study also offers several notable strengths that enhance the relevance and practical value of its findings. First, we included participants from diverse cultural backgrounds to reflect the composition of the Dutch patient population. Children from immigrant families and those living in asylum seeker centers are often underrepresented in research. With support of a social worker and a translator, we were able to include this group of participants. Considering cultural factors is essential in the management of IMDs [56]. Second, we developed the semi-structured interview guide in collaboration with colleagues from different medical disciplines and roles within the team. This multidisciplinary input ensured that the interviews addressed a broad spectrum of relevant topics.

While analyzing the themes, metabolic pediatricians noted that several problems and experiences described in the interviews had not previously emerged during clinical consultations. A possible explanation is that people with IMD and their caregivers may experience a barrier to discussing personal or private matters with their doctors, or may simply not realize that sharing such information could be important. Conversely, some challenges sometimes encountered in the consultation room did not emerge in our interviews, such as difficulties related to relationships, divorced families and living in asylum seekers' centers.

It is important to note that, in addition to the IMD and its nutritional treatment, other factors – such as marital status, socioeconomic status and cultural backgrounds – also influence the burden of illness and levels of psychological distress or resilience.

Cognitive and emotional coping styles, perinatal traumatic stress, and family problems all influence social-emotional outcome. For example, divorce or strained relationships have been linked to poorer metabolic control and reduced parental involvement in nutritional compliance [57], [58].

To gain more insight into the emotional and behavior dimension, using a questionnaire such as for example the Child Behavior Checklist (CBCL) could be relevant. Jusiene and Kucinskas [59] used this instrument and found that children with PKU were more withdrawn, more anxious or depressed and experienced greater social and attention problems. These problems were related to parental feelings of guilt and anger, as well as maladaptive stress coping strategies. Expanding our understanding of these issues in people with IMDs more broadly would be highly valuable. In this context, it is especially valuable to specifically address these issues in studies on (very) rare IMDs with acute risk profiles, given the scarcity of research on this topic—particularly in comparison to PKU, for which far more studies have been conducted and multiple systematic reviews are already available.

The relationship between IMDs, nutritional treatment and social-emotional functioning remains insufficiently understood, largely because this dimension has received limited attention in both clinical practice and research. As a result, psychosocial services for people with IMD and their caregivers are often lacking or not readily accessible. This gap is further compounded by long waiting lists for mental health care. Importantly, stress does not arise solely from the demands of nutritional therapy; psychological distress may already begin at the moment of diagnosis [60], [61].

Patients and families who are experiencing difficulties can be helped through psychoeducation, for example on different parenting styles. In a series of articles, Sebri et al. have described how psychological interventions and e-health applications can be part of the care provided to people with chronic illnesses [62], [63], [64]. Part of these difficulties may be resolved or reduced by actively incorporating the findings of this study and other research in new technological applications, such as *E*-health and self-managing tools for patients. Gee et al. presented a theoretical framework (eHealth Enhanced Chronic Care Model) which has been shown to improve health outcomes of chronically ill patients [65].

In the future, psychological interventions may be used to improve the quality of life for patients and their families. For example, cognitive behavioral therapy can help patients with chronic illnesses [66]. Other treatments can also be considered, such as COMET (Competitive Memory Training) to improve self-image, or ACT (Acceptance and Commitment Therapy) to learn to accept negative thoughts and emotions.

We also know that peer support and the exchange of experiences are highly valued by families. Facilitating opportunities for structured peer contact, alongside routine assessment of social-emotional and psychosocial functioning, would therefore be an important step toward improving care. The findings of our study can support physicians and other healthcare professionals in providing age-appropriate guidance related to nutritional treatment. Because different challenges arise at different developmental stages, the timeline presented in Fig. 2 may serve as a practical tool to guide clinical conversations and tailor support across the lifespan.

**Fig. 2:**
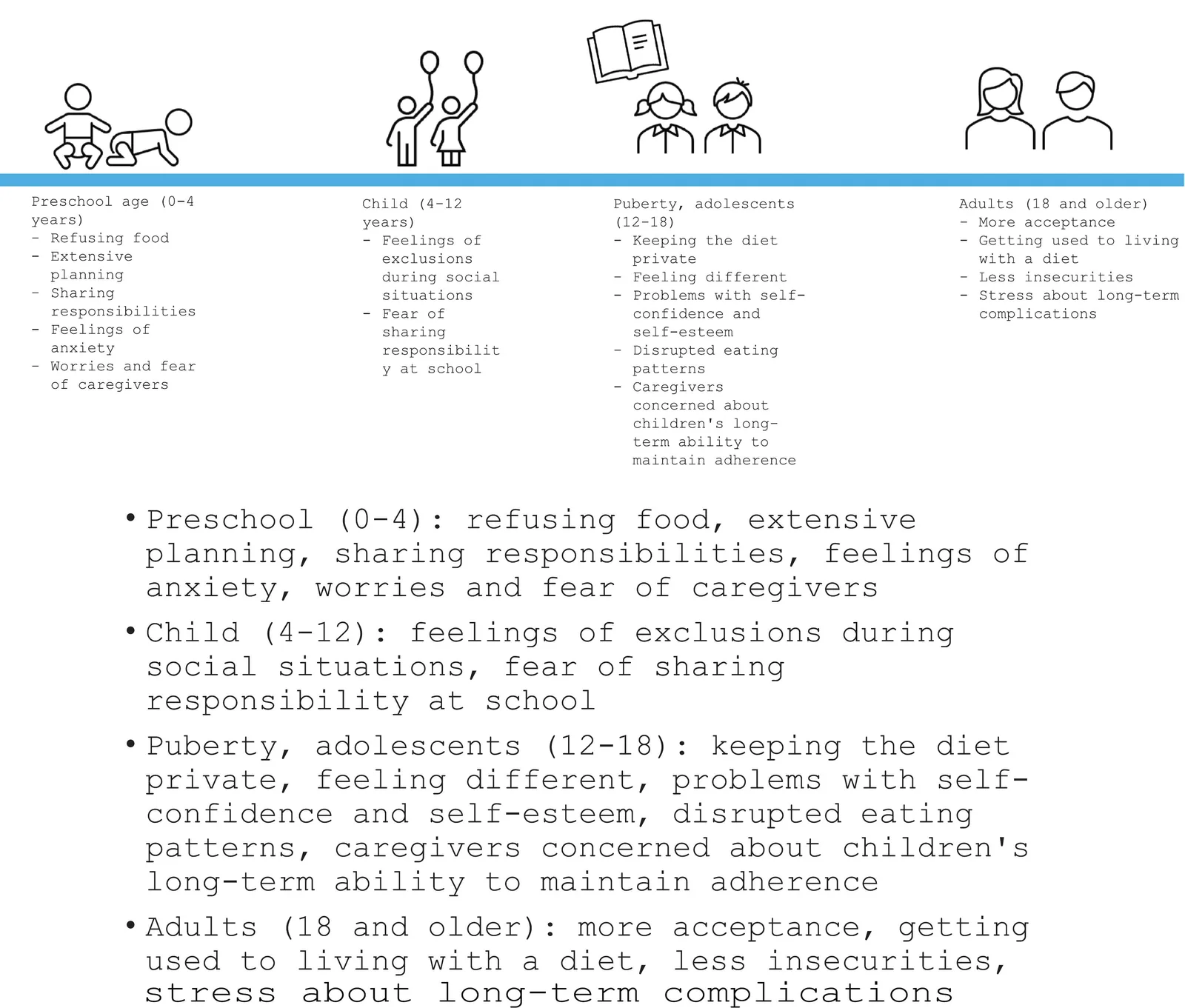
Different challenges at different developmental stages.

In conclusion, nutritional treatment in IMDs is often burdensome, affecting daily life and psychological well-being through ongoing inconveniences, social restrictions, and reduced spontaneity. Although many individuals gradually develop coping strategies and reach a level of acceptance, the burden of diet is also shaped by broader factors such as socio-emotional difficulties and perinatal stress or trauma, underscoring the need for comprehensive support for people with IMD and their caregivers.

## Contribution of individual authors

FKW, ABH-S and FJvS initiated this project. AV, FKW and ABH-S were involved in study design, data collection, data analysis and wrote the first and final version of the manuscript. FdB, RMC-B TGJD, MJF-W, YH, CMAL and FJvS were involved in data analysis and revised the manuscript for important intellectual content. DDvV and MMvdK revised the manuscript for important intellectual content. All authors approved the final manuscript as submitted and agreed to be accountable for all aspects of the work. All authors confirm the absence of previous similar or simultaneous publications.

## Ethics approval and patient consent statement

The Medical Ethical Review Committee of UMCG concluded that this study fell outside the scope of the Dutch Medical Research Involving Human Subjects Act. Informed consent was obtained from patients and/or caregivers before the interviews, and the participants had the right to end their participation at any time.

## CRediT authorship contribution statement

**Annieke Venema:** Writing – original draft, Methodology, Formal analysis. **Frederiec K. Withaar:** Writing – original draft, Methodology, Formal analysis, Conceptualization. **Foekje de Boer:** Writing – review & editing. **Rianne M. Cnossen- Bosma:** Writing – review & editing, Project administration. **Terry G.J. Derks:** Writing – review & editing. **Danique Draaisma- van Vliet:** Writing – review & editing. **Marieke J. Fokkert-Wilts:** Writing – review & editing, Data curation. **Yteke Hoekstra:** Writing – review & editing, Data curation. **Melanie M. van der Klauw:** Writing – review & editing. **Charlotte M.A. Lubout:** Writing – review & editing. **Francjan J. van Spronsen:** Writing – review & editing, Conceptualization. **Andrea B. Haijer-Schreuder:** Writing – review & editing, Writing – original draft, Supervision, Methodology, Formal analysis, Conceptualization.

## Funding

The MD-PhD scholarship of AV (MD-PhD 21–65) is funded by the Junior Scientific Masterclass from the University of Groningen, University Medical Center Groningen; FKW received a grant from the Foundation Friends of Beatrix Children's Hospital UMCG.

## Declaration of competing interest

ABH-S declares that she experiences no competing interests concerning the content of this manuscript. However, there are confidentiality agreements with third parties. For all private-public relationships, all contracts are via UMCG Contract Research Desk and all payments are to UMCG.

TGJD declares that she experiences no competing interests concerning the content of this manuscript. However, there are confidentiality agreements with third parties. For all private-public relationships, all contracts are via UMCG Contract Research Desk and all payments are to UMCG.

FJvS declares that he experiences no competing interests concerning the content of this manuscript. However, there are agreements with third parties on advisory role, participation in advisory boards and data safety monitoring board, and giving lectures/chairing roles. These parties include Agios, AlltRNA, Applied Pharma Research, Arla Food Int, BioMarin, Maze, Moderna, Nutricia, Pluvia Biotech, PTC Therapeutics, Travere, Ultragenyx and Vitoflo, while there are also grants from ESPKU, NPKUA, NPKUV, Nutricia, Sanofi, Sobi, and Tyrosinemia Foundation. For all private-public relationships, all contracts are via UMCG Contract Research Desk and all payments are to UMCG. All other authors declare no conflicts of interest.

All other authors declare that they have no competing interests.

## Data Availability

The data that support the findings of this study are available from the corresponding author upon reasonable request.
