# Supplementary material for: The psychological burden of nutritional treatment in inherited metabolic disorders: Navigating daily life
Source: Mol Genet Metab Rep. 2026 Sep 25;49:101365. doi: 10.1016/j.ymgmr.2026.101365 (PMC13635391; doi:10.1016/j.ymgmr.2026.101365)
Supplement: Supplementary file 1 [file mmc1.docx]

**Supplementary File 1. Interview Format**

*Structure of the semi‑structured interview*

**Bold questions:** main questions
Light‑printed questions: themes and follow‑up questions if these have not yet been addressed

------------------------------------------------------

**How do you organise eating moments, at home and outside the home? Can you briefly explain this?**

(Think of breakfast, lunch, dinner, snacks, preparing supplements/cornstarch, night-time feeding, lunch and snacks at school/work, treats, preparing and taking supplements with you, night-time feeding, eating out together, parties, outings.)

Why does this work well for you?

What problems or difficulties do you encounter in this area?

**What problems or difficulties do you encounter during periods of illness/use of the emergency protocol?**

Have there been events related to the dietary treatment that influence the current situation? (e.g., anxiety?)

------------------------------------------------------

**What limitations do you experience due to the dietary treatment, or which situations do you avoid?**

(Why do you experience this? What is the impact on your social life?)

**In which situations are you able to adhere to the dietary treatment, and in which situations not?**

(Why yes and why not? Is the dietary treatment sustainable? What concerns do you have or not have? In what ways do you have influence over the dietary treatment?)

**What responsibilities do you feel for yourself, or do others feel, regarding the dietary treatment?**

(Is this responsibility shared by others in your network, e.g., school? Are there also people in your environment who do not take the dietary treatment seriously or do not recognise its impact?)

**Does the dietary treatment influence how well you sleep?**

(Why does sleeping go well or not well?)

------------------------------------------------------

**In what ways does the dietary treatment influence your satisfaction with life?**

**In what ways does the dietary treatment influence how you feel?**

(Think of mood, stress, anxiety—what are you afraid of—sadness, anger, worries about the future such as independence, letting go versus control.)

**In what ways does the dietary treatment influence your self-image and self-confidence?**

(Do you feel different from others?)

**Have you or one of your family members needed professional help because of consequences of the dietary treatment?**

------------------------------------------------------

**Which question do you think should always be asked during an outpatient clinic appointment?**

**What has the dietary treatment taught or brought you?**

(What are the qualities/strengths of your child/your family in dealing with the dietary treatment?)

**Do you have any further questions?**

**Is there a topic that has not yet been discussed?**

------------------------------------------------------

**For people who no longer have dietary treatment:**

What was the impact of your diet, now that you can compare it with your current situation without dietary treatment?
