# Supplementary material for: The psychological burden of nutritional treatment in inherited metabolic disorders: Navigating daily life
Source: Mol Genet Metab Rep. 2026 Sep 25;49:101365. doi: 10.1016/j.ymgmr.2026.101365 (PMC13635391; doi:10.1016/j.ymgmr.2026.101365)
Supplement: Supplementary file 2 [file mmc2.docx]

Supplementary file 2. Overview of research articles for systematic review

|  | **Author (Year)** | **Population** | **Study design/methods** | **Summary of impact nutritional treatment** |
| --- | --- | --- | --- | --- |
| **PKU** | | | | |
| 1 | Maissen-Abgottspon (2023)(1) | Adults | Questionnaire: PKU-QOL | Most patients reported no or little overall burden from PKU or dietary protein restriction, though a notable subgroup experienced moderate–major impact, with higher Phe levels—but not age, sex, or IQ—associated with greater perceived burden. |
| 2 | Morawska (2020)(2) | Children | Questionnaires | Parental guilt over poor dietary adherence showed the highest burden, with additional moderate impact reflected in emotional strain, anxiety about Phe levels, and practical challenges of dietary protein restriction. |
| 3 | Becsei (2021)(3) | Children | Questionnaires | Although most domains showed little QoL impact, dietary protein restriction had the greatest burden—especially reduced food enjoyment—while adherence and social impact were minimal, and even well‑compliant adolescents reported moderate emotional/taste burdens and severe impact on food enjoyment. |
| 4 | Evans (2019)(4) | Children; parents | Questionnaires (1) mealtime emotions, (2) feed-time, (3) Beck’s anxiety inventory and (4) the child health inventory for parents (CHIP) | Mealtime stress and mood were generally positive and similar across groups, though families with PKU showed a trend toward slightly less enjoyable mealtimes. |
| 5 | Alptekin (2018)(5) | Patients; 95,3% on a diet, 17,4% on BH4 | PKU-QOL | Children showed generally high burden scores—especially guilt and reduced food enjoyment—while overall difficulty with dietary restriction was lower, and similar patterns across groups suggest that PKU‑related diet rules and supplements can also create social challenges. |
| 6 | Bosch (2015)(6) | Patients; parents | HRQoL generic measures, PKU-QOL | Emotional strain, anxiety about Phe levels, and guilt over poor dietary or supplement adherence showed the greatest PKU‑QOL impact, while individuals with mild/moderate PKU or on BH4 reported notably lower practical and emotional burden |
| 7 | Carpenter (2018)(7) | Parents of children <2 years of age | Interviews | Parents described substantial emotional and practical burden—including fear of consequences of non‑compliance, heightened responsibility, guilt, frustration, anxiety, and concern about social judgment—closely mirroring themes found in our own interviews. |
| 8 | Di Ciommo (2012)(8) | Children; adolescents; young adults | Semi-structured interviews | Living with PKU requires a demanding lifelong diet, with unpalatable special foods and a strong sense of ‘being different,’ yet patients generally maintain good autonomy in managing these restrictions. |
| 9 | Zwiesele (2015)(9) | Children; parents | Interviews, qualitative | Parents reported that children most often felt ‘different’ during school events and social gatherings, with additional triggers including general food situations, peer reactions, family events, and irritability related to dietary control. |
| 10 | Awiszus (1990)(10) | Parents | Narrative interviews | Parents and patients described wide‑ranging challenges—including learning and managing the diet, practical and financial burdens, balancing restrictions, and the ongoing process of accepting the dietary demands. |
| 11 | Vegni (2010)(11) | Patients | In-depth interviews | The diet was experienced as both positive and negative, becoming an obstacle when publicly visible—making the disease ‘seen’ during eating—and across age groups families described the social exposure and growing responsibility involved in managing dietary demands. |
| 12 | Klimek (2020)(12) | Adults, 82% adhere to a diet | Online survey | Diet interruption was mainly driven by dietary restrictions, lifestyle and work conflicts, time demands, and cost, while most patients felt better on diet and those maintaining a Phe‑calculated diet showed markedly lower—though not statistically significant—rates of depressive symptoms compared with those not following strict dietary control. |
| 13 | Diesen (2016)(13) | Young adults | In-depth interviews | Adults with PKU viewed the demanding diet as essential for preserving cognitive health, describing lifelong hard work, early resistance, and later deep appreciation for the strict, unwavering parental commitment that safeguarded their development. |
| 14 | Luu (2020)(14) | Adolescents; adults | Survey | Patients on a restricted‑protein diet reported more frequent preoccupation with food, though maladaptive eating attitudes were generally uncommon. |
| 15 | Antisdel (2000)(15) | Adolescents; young women | Self-report questionnaires; The Eating Attitudes Test-26, Eating Disorder Inventory, The Coopersmith Self-Esteem Inventory, The Body Cathexis Scale and more | Marked disordered‑eating symptoms were reported in a minority of females with diabetes (33%) and PKU (23%), with no significant group difference due to differing assessment scales. |
| 16 | Sharman (2013)(16) | Adolescents | Focus group | Participants highlighted multiple barriers to dietary adherence—including explaining PKU to others, managing and storing formula, resisting tempting foods, finding suitable options in restaurants, and interpreting food labels—alongside several themes describing what helped them stay adherent. |
| 17 | Zengin Akkus (2020)(17) | Parents; 48 with diet restrictions, 41 without diet restrictions | Semi-structured interviews, questionnaires | Parents of children with dietary restrictions showed significantly higher depressive symptom scores, although rates of anxiety and clinically elevated symptoms did not differ between the groups. |
| 18 | Bilginsoy  (2005)(18) | Patients; family | Survey | Although most caretakers were knowledgeable about the diet and did not find its preparation burdensome, dissatisfaction—especially with the limited variety of PKU foods—was widespread, with unhappy caretakers and children outnumbering satisfied ones by two to one. |
| 19 | Haitjema (2022)(19) | Caregivers | Web-based questionnaire; behavioral pediatrics feeding assessment scale, PKU-specific questionnaire | Caregivers of children with PKU reported greater difficulty providing food variety, more stress during meals outside the home, stricter reactions to food‑related mishaps, and higher levels of anger, frustration, and anxiety during feeding, along with more concern about negative health effects of their child’s eating pattern. |
| 20 | Ievers-Landis  (2005)(20) | Caregivers; children; adolescents | Semi-structured interviews | Diet‑related problems were reported by most caregivers (84%) and all children, including nonadherence, social discomfort around formula use, scheduling and planning difficulties, negative feelings about the diet, and gaps in knowledge. |
| 21 | Medford (2017)(21) | Mothers | Measure; Child Dependency Score | A mean care‑dependency score of 5.78 indicated that children were highly reliant on caregivers for maintaining dietary treatment adherence. |
| 22 | Roberts (2014)(22) | Women | Interviews | Women with PKU—especially those planning pregnancy—were deeply concerned about maintaining low Phe levels, and mothers described major social and practical burdens such as avoiding food‑centred gatherings, weighing food wherever they ate, and always carrying supplements. |
| 23 | Afifi (2022)(23) | Children; parents | PKU-QOL questionnaire | Children under five on a special diet and supplements showed higher physical, emotional, and total scores—though only physical functioning was significantly affected—while many parents reported sadness related to dietary restrictions but relatively little daily‑life impact from supplement disputes or protein‑weighing routines. |
| 24 | Lord (2005)(24) | Parents | Questionnaires | On the PKU Checklist, all items caused at least some concern for parents, with the greatest worry—shared by both mothers and fathers—being whether the diet would be followed when they were not present to supervise. |
| 25 | Iakovou (2020)(25) | ? | Questionnaire about self-esteem | Self‑esteem was highest among patients who adhered strictly to the PKU diet, while those with partial or poor adherence more often reported low or moderate self‑esteem—linked to guilt, limited understanding of the diet’s benefits, or lack of acceptance of the role of low Phe levels in academic and daily functioning. |
| 26 | Das (2014)(26) | Adults, adolescents | Hr-QOL | Quality of life and mood did not differ significantly across dietary groups, and patients on a strict protein‑restricted diet with amino‑acid supplementation reported feeling no worse than those on a normal diet. |
| 27 | Diesen (2015)(27) | Young adults | Semi-structured interviews | The diet was described as both demanding and deeply identity‑shaping, with adults explaining that lifelong dietary vigilance influenced their daily routines, social interactions, and sense of self, while also fostering strong appreciation for the diet’s role in protecting their cognitive development. |
| **IMD** | | | | |
| 1 | Ouattara (2022)(28) | Children with a restrictive diet | Questionnaires | Structural equation modelling showed that earlier diagnosis and the potential need for an emergency diet were linked to better and poorer child QoL respectively, while enteral feeding was associated with greater child difficulties; the emergency diet also had a significant positive indirect effect on QoL through reduced child trait anxiety, with a smaller pathway additionally involving parent anxiety. |
| 2 | Bosch (2021)(29) | Children and parents: Intoxication-type IEM; 85 acute patients (64.7% with current dietary treatment) and 90 PKU patients (83.3% with current dietary treatment) | Analysis of international data set | Dietary treatment at the time of assessment was associated with significantly lower overall, physical, psychosocial, social, and school functioning scores, and regression analyses suggested that reduced HrQoL likely reflects the combined burden of the disease itself and the demands of dietary management. |
| 3 | Shaji-Thomas (2017)(30) | Parents | Explorative descriptive survey design; The RAND 36-Item Health Survey version 1 | HRQoL was significantly associated with certain demographic factors, with children whose caregivers prepared a special diet showing notably different general‑health scores, indicating that dietary preparation demands were linked to variations in perceived health. |
| 4 | Siddiq (2016)(31) | Caregivers | Semi-structured interviews | Diet‑related themes included adapting to a ‘new normal’ through proactive coping, navigating social stressors, and strong parental advocacy, alongside additional themes focused on families’ broader experiences with healthcare and support. |
| 5 | Gramer (2014)(32) | Caregivers of children with IMD, detected with newborn screening | Questionnaire | Among children receiving treatment, dietary management was linked to higher odds of families experiencing at least a moderate burden compared with those using medical treatment alone. |
| 6 | Dimitrova (2021)(33) | Youth | Questionnaires: HR-QoL, The Strengths and Difficulties Questionnaire, The Cognitive Emotional Regulation Questionnaire, Pediatric Inventory for Parents, Hospital anxiety and depression scale. Diet as covariate | At baseline, dietary treatment showed no significant effects on any child or parent QoL dimensions. |
| 7 | Kemper (2010)(34) | Women at summer camp (PKU, MMA, MSUD) | Focus group | Participants described strong sensory dislike of medical and low‑protein foods and social challenges at school, while themes influencing dietary adherence included insurance barriers, temptation to deviate from the diet, and reliance on support from friends and family. |
| 8 | Harings (2024)(35) | Parents | Survey | Nutritional treatment imposed a considerable daily burden on families, with strict dietary requirements affecting routines, social participation, and caregiver stress. |
| 9 | Evans (2012)(36) | Children with IEM; UCD, OA, and amino acid disorders (except PKU) | Observational, questionnaire | Children showed multiple feeding difficulties, including poor appetite, limited food variety, prolonged mealtimes, vomiting, and negative mealtime behaviours, with many experiencing more than one difficulty and reduced engagement in the social aspects of eating. |
| 10 | Rajasekar (2020)(37) | Parents | Semi-structured interviews | The diet was described as burdensome, with families facing challenges in adherence, meal preparation, and maintaining metabolic control as part of daily life. |
| 11 | Schnabel-Besson  (2025)(38) | Children with IMD detected by NBS and their parents | Questionnaires | Parents of children requiring strict dietary treatment reported substantially higher burden—often at least moderate and more frequently severe—than those without dietary treatment, with no clear differences between the various diet types. |
| 12 | Yamaguchi (2016)(39) | Patients | Literature review | Across infancy, school age, and adolescence, diet‑related demands contributed to parental anxiety, children’s social stress and comparison with peers, and later medical, economic, and social difficulties, with overall QoL reduced for both children and caregivers despite symptom control. |
| **Tyrosinemia type I** | | | | |
| 1 | Campbell (2018)(40) | Caregivers | Questionnaire TYR-QOL | Most caregivers reported strong feelings of guilt around dietary or formula nonadherence, and many experienced moderate to severe quality‑of‑life impacts from managing both the strict diet and the specialized formula. |
| 2 | Malik (2015)(41) | Adolescents; families | Mixed-Method Investigation: Qualitative interviews; quantitative survey | The study reported that the strict dietary regimen contributed to increased psychological strain, with affected individuals showing higher levels of stress, worry, and emotional burden linked to the demands and restrictions of the diet |
| **Galactosemia** | | | | |
| 1 | Hoffmann (2012)(42) | Adults | Questionnaires, ‘Quality of Life Profile of the chronically ill’ (PLC) | While most patients (58,5%) reported no difficulty keeping the diet, about one‑third found it at least partly burdensome, and greater perceived dietary burden was linked to more negative mood and lower positive mood, social wellbeing, and social functioning. |
| 2 | Lambert (2004)(43) | Patients | Interviews | For people with galactosaemia, the most distressing aspect of the condition was the immediate inconvenience and disruption caused by strict food restrictions. |
| **Long-chain fatty acid oxidation disorders** | | | | |
| 1 | Williams-Hall (2022)(44) | Caregivers; patients | Focus group, semi-structured interviews, interviews expert clinicians | Patients and caregivers reported far more diet‑related QoL impacts than clinicians recognized, including reduced spontaneity, increased planning and caregiver dependence, limitations in social participation and relationships, disruptions to daily activities such as self‑care and travel, and emotional burdens such as stress, helplessness, and isolation. |
| 2 | Thiel (2023)(45) | Families | Web-based questionnaire | Caregivers—especially mothers—reported substantial involvement and stress related to managing their child’s special diet, with sleep disruption, challenges eating outside the home, variable difficulty during early childhood, and a need to prepare meals for most settings, although most parents felt they maintained adequate dietary adherence. |
| **MSUD** | | | | |
| 1 | Packman (2012)(46) | Young adults | Semi-structured interviews | Young adults with MSUD described the diet as the most difficult aspect of the condition, highlighting the lifelong strain of strict dietary management and the frustration of not being able to fit in socially with peers. |
| 2 | Packman (2007)(47) | Families | MSUD Family Survey; questionnaires | Parents reported that MSUD significantly affected daily life and career decisions, leaving them with less time for their other children and themselves due to the substantial time spent managing dietary requirements. |
| **Familial chylomicronemia syndrome** | | | | |
| 1 | Williams (2023)(48) | Patients | Interviews | Participants described profound social and practical burdens from their highly restricted diet, including difficulty socializing, avoiding food‑related events, low satisfaction with a monotonous and fear‑laden diet, constant monitoring and planning, limited flexibility, challenges eating outside the home or with family, and reliance on close relatives to help maintain strict dietary control. |
| **GSD** | | | | |
| 1 | Venema (2021)(49) | Patients with hepatic GSD and IKH | Retrospective, health records | A substantial proportion of patients showed avoidant or restrictive eating patterns—marked by poor appetite, selective and slow eating, and aversive mealtime symptoms—alongside frequent sleep problems and high parental stress related to the child’s illness. |
| 2 | Flanagan (2015)(50) | Patients | Quantitative and qualitative | Although patients reported fewer classic eating‑disorder symptoms than population norms, they showed elevated dysfunctional attitudes toward food and lower body esteem. |
| **Lysosomal acid lipase deficiency** | | | | |
| 1 | Hassall (2022)(51) | Parents | Semi-structured interviews | Parents described eating and symptom control as central to their child’s ability to live a normal life, with many viewing the restricted diet as manageable and relatively normal, while others struggled with the social and emotional challenge of their child being unable to eat the same foods as the family. |
| **Urea cycle disorders** | | | | |
| 1 | Cederbaum (2001)(52) | Families | 30-item questionnaire developed, to provide understanding of the stresses imposed by the occurence of UCD in a family | Families reported a wide range of burden associated with obtaining special foods and medicines, with responses clustering at both low and high levels, indicating that while some experienced minimal hassle, others found the task considerably burdensome. |

**References**

1.	Maissen-Abgottspon S, Muri R, Hochuli M, Reismann P, Barta AG, Alptekin IM, et al. Health-related quality of life in a european sample of adults with early-treated classical PKU. Orphanet J Rare Dis. 2023;18(1):300.

2.	Morawska A, Mitchell AE, Etel E, Kirby G, McGill J, Coman D, Inwood A. Psychosocial functioning in children with phenylketonuria: Relationships between quality of life and parenting indicators. Child Care Health Dev. 2020;46(1):56-65.

3.	Becsei D, Hiripi R, Kiss E, Szatmari I, Arato A, Reusz G, et al. Quality of life in children living with PKU - a single-center, cross-sectional, observational study from Hungary. Mol Genet Metab Rep. 2021;29:100823.

4.	Evans S, Daly A, Wildgoose J, Cochrane B, Ashmore C, Kearney S, MacDonald A. Mealtime Anxiety and Coping Behaviour in Parents and Children During Weaning in PKU: A Case-Control Study. Nutrients. 2019;11(12).

5.	Alptekin IM, Koc N, Gunduz M, Cakiroglu FP. The impact of phenylketonuria on PKU patients' quality of life: Using of the phenylketonuria-quality of life (PKU-QOL) questionnaires. Clin Nutr ESPEN. 2018;27:79-85.

6.	Bosch AM, Burlina A, Cunningham A, Bettiol E, Moreau-Stucker F, Koledova E, et al. Assessment of the impact of phenylketonuria and its treatment on quality of life of patients and parents from seven European countries. Orphanet J Rare Dis. 2015;10:80.

7.	Carpenter K, Wittkowski A, Hare DJ, Medford E, Rust S, Jones SA, Smith DM. Parenting a Child with Phenylketonuria (PKU): an Interpretative Phenomenological Analysis (IPA) of the Experience of Parents. J Genet Couns. 2018;27(5):1074-86.

8.	Di Ciommo V, Forcella E, Cotugno G. Living with phenylketonuria from the point of view of children, adolescents, and young adults: a qualitative study. J Dev Behav Pediatr. 2012;33(3):229-35.

9.	Zwiesele S, Bannick A, Trepanier A. Parental strategies to help children with phenylketonuria (PKU) cope with feeling different. Am J Med Genet A. 2015;167A(8):1787-95.

10.	Awiszus D, Unger I. Coping with PKU: results of narrative interviews with parents. Eur J Pediatr. 1990;149 Suppl 1:S45-51.

11.	Vegni E, Fiori L, Riva E, Giovannini M, Moja EA. How individuals with phenylketonuria experience their illness: an age-related qualitative study. Child Care Health Dev. 2010;36(4):539-48.

12.	Klimek A, Baerwald C, Schwarz M, Rutsch F, Parhofer KG, Plockinger U, et al. Everyday Life, Dietary Practices, and Health Conditions of Adult PKU Patients: A Multicenter, Cross-Sectional Study. Ann Nutr Metab. 2020;76(4):251-8.

13.	Diesen PS. "I Feel Lucky" - Gratitude Among Young Adults with Phenylketonuria (PKU). J Genet Couns. 2016;25(5):1002-9.

14.	Luu S, Breunig T, Drilias N, Kuhl A, Scott Schwoerer J, Cody P. A Survey of Eating Attitudes and Behaviors in Adolescents and Adults With Phenylalanine Hydroxylase Deficiency. WMJ. 2020;119(1):37-43.

15.	Antisdel JE, Chrisler JC. Comparison of eating attitudes and behaviors among adolescent and young women with type 1 diabetes mellitus and phenylketonuria. J Dev Behav Pediatr. 2000;21(2):81-6.

16.	Sharman R, Mulgrew K, Katsikitis M. Qualitative analysis of factors affecting adherence to the phenylketonuria diet in adolescents. Clin Nurse Spec. 2013;27(4):205-10.

17.	Zengin Akkus P, Bilginer Gurbuz B, Ciki K, Ilter Bahadur E, Karahan S, Ozmert EN, et al. Caring for a Child with Phenylketonuria: Parental Experiences from a Eurasian Country. J Dev Behav Pediatr. 2020;41(3):195-202.

18.	Bilginsoy C, Waitzman N, Leonard CO, Ernst SL. Living with phenylketonuria: perspectives of patients and their families. J Inherit Metab Dis. 2005;28(5):639-49.

19.	Haitjema S, Lubout CMA, Abeln D, Bruijn-van der Veen M, MacDonald A, Wolffenbuttel BHR, van Spronsen FJ. Dietary treatment in Dutch children with phenylketonuria: An inventory of associated social restrictions and eating problems. Nutrition. 2022;97:111576.

20.	Ievers-Landis CE, Hoff AL, Brez C, Cancilliere MK, McConnell J, Kerr D. Situational analysis of dietary challenges of the treatment regimen for children and adolescents with phenylketonuria and their primary caregivers. J Dev Behav Pediatr. 2005;26(3):186-93.

21.	Medford E, Hare DJ, Carpenter K, Rust S, Jones S, Wittkowski A. Treatment Adherence and Psychological Wellbeing in Maternal Carers of Children with Phenylketonuria (PKU). JIMD Rep. 2017;37:107-14.

22.	Roberts RM, Muller T, Sweeney A, Bratkovic D, Gannoni A. Promoting psychological well-being in women with phenylketonuria: Pregnancy-related stresses, coping strategies and supports. Mol Genet Metab Rep. 2014;1:148-57.

23.	Afifi ZEM SS, Eldaleel MMA, Abd El Fatah SAM. Quality of life of children with phenylketonuria at primary health care center in Cairo Egypt: An exploratory cross-sectional study. Vulnerable Children and Youth Studies. 2023;18:2:282-97.

24.	Lord B WC, Ungerer J. Parent Reactions to Childhood Phenylketonuria. Families, Systems, & Health. 2005;23(2):204–19.

25.	Iakovou KK, Schulpis K. Self-estimation of phenylketonuria patients on therapeutic diet. Psychological support. Drug Metab Pers Ther. 2020;35(2).

26.	Das AM, Goedecke K, Meyer U, Kanzelmeyer N, Koch S, Illsinger S, et al. Dietary habits and metabolic control in adolescents and young adults with phenylketonuria: self-imposed protein restriction may be harmful. JIMD Rep. 2014;13:149-58.

27.	Plata Sofie Diesen IW, Lisbet Grut & Bengt Frode Kase Betwixtand between being healthy and ill: the stigma experienced by young adults with phenylketonuria. Scandinavian Journal of Disability Research. 2014;17(4):321-334.

28.	Ouattara A, Resseguier N, Cano A, De Lonlay P, Arnoux JB, Brassier A, et al. Determinants of Quality of Life in Children with Inborn Errors of Metabolism Receiving a Restricted Diet. J Pediatr. 2022;242:192-200 e3.

29.	Bosch F, Landolt MA, Baumgartner MR, Zeltner N, Kolker S, Gleich F, et al. Health-related quality of life in paediatric patients with intoxication-type inborn errors of metabolism: Analysis of an international data set. J Inherit Metab Dis. 2021;44(1):215-25.

30.	Shaji Thomas D, Mohd Wali Shakman L, Saraswathy K, Arulappan J. Parenting a child with metabolic diseases: Impact on health related quality of life of parents. Diabetes Metab Syndr. 2017;11(1):25-9.

31.	Siddiq S, Wilson BJ, Graham ID, Lamoureux M, Khangura SD, Tingley K, et al. Experiences of caregivers of children with inherited metabolic diseases: a qualitative study. Orphanet J Rare Dis. 2016;11(1):168.

32.	Gramer G, Haege G, Glahn EM, Hoffmann GF, Lindner M, Burgard P. Living with an inborn error of metabolism detected by newborn screening-parents' perspectives on child development and impact on family life. J Inherit Metab Dis. 2014;37(2):189-95.

33.	Dimitrova N, Glaus J, Urben S, Wuthrich V, Morisod Harari M, Ballhausen D. The impact of disease severity on the psychological well-being of youth affected by an inborn error of metabolism and their families: A one-year longitudinal study. Mol Genet Metab Rep. 2021;29:100795.

34.	Kemper AR, Brewer CA, Singh RH. Perspectives on dietary adherence among women with inborn errors of metabolism. J Am Diet Assoc. 2010;110(2):247-52.

35.	Harings T, Neininger MP, Eisenhofer S, Thiele AG, Kiess W, Bertsche A, et al. The impact of a child's inborn error of metabolism: the parents' perspectives on restrictions, discrimination, family planning, and emergency management. Orphanet J Rare Dis. 2024;19(1):313.

36.	Evans S, Alroqaiba N, Daly A, Neville C, Davies P, Macdonald A. Feeding difficulties in children with inherited metabolic disorders: a pilot study. J Hum Nutr Diet. 2012;25(3):209-16.

37.	Rajasekar P, Gannavarapu S, Napier M, Prasad AN, Vasudev A, Mantulak A, et al. Parental psychosocial aspects and stressors involved in the management of inborn errors of metabolism. Mol Genet Metab Rep. 2020;25:100654.

38.	Schnabel-Besson E, Garbade SF, Gleich F, Grunert SC, Kramer J, Thimm E, et al. Parental and child's psychosocial and financial burden living with an inherited metabolic disease identified by newborn screening. J Inherit Metab Dis. 2025;48(1):e12784.

39.	Yamaguchi K, Wakimizu R, Kubota M. Difficulties in Daily Life and Associated Factors, and QoL of Children with Inherited Metabolic Disease and Their Parents in Japan: A Literature Review. JIMD Rep. 2017;33:1-10.

40.	Campbell H, Singh RH, Hall E, Ali N. Caregiver Quality of Life with Tyrosinemia Type 1. J Genet Couns. 2018;27(3):723-31.

41.	Malik S, NiMhurchadha S, Jackson C, Eliasson L, Weinman J, Roche S, Walter J. Treatment Adherence in Type 1 Hereditary Tyrosinaemia (HT1): A Mixed-Method Investigation into the Beliefs, Attitudes and Behaviour of Adolescent Patients, Their Families and Their Health-Care Team. JIMD Rep. 2015;18:13-22.

42.	Hoffmann B, Dragano N, Schweitzer-Krantz S. Living situation, occupation and health-related quality of life in adult patients with classic galactosemia. J Inherit Metab Dis. 2012;35(6):1051-8.

43.	Lambert C, Boneh A. The impact of galactosaemia on quality of life--a pilot study. J Inherit Metab Dis. 2004;27(5):601-8.

44.	Williams-Hall R, Tinsley K, Kruger E, Johnson C, Bowden A, Cimms T, Gater A. Qualitative evaluation of the symptoms and quality of life impacts of long-chain fatty acid oxidation disorders. Ther Adv Endocrinol Metab. 2022;13:20420188211065655.

45.	Thiel M, Garbade SF, Rosenbaum-Fabian S, Spiekerkoetter U, Grunert SC. Psychosocial issues and coping strategies in families affected by long-chain fatty acid oxidation disorders. JIMD Rep. 2024;65(1):25-38.

46.	Packman W, Mehta I, Rafie S, Mehta J, Naldi M, Mooney KH. Young adults with MSUD and their transition to adulthood: psychosocial issues. J Genet Couns. 2012;21(5):692-703.

47.	Packman W, Henderson SL, Mehta I, Ronen R, Danner D, Chesterman B, Packman S. Psychosocial issues in families affected by maple syrup urine disease. J Genet Couns. 2007;16(6):799-809.

48.	Williams K, Tickler G, Valdivielso P, Alonso J, Vera-Llonch M, Cubells L, Acaster S. Symptoms and impacts of familial chylomicronemia syndrome: a qualitative study of the patient experience. Orphanet J Rare Dis. 2023;18(1):316.

49.	Venema A, Peeks F, de Bruijn-van der Veen M, de Boer F, Fokkert-Wilts MJ, Lubout CMA, et al. A retrospective study of eating and psychosocial problems in patients with hepatic glycogen storage diseases and idiopathic ketotic hypoglycemia: Towards a standard set of patient-reported outcome measures. JIMD Rep. 2022;63(1):29-40.

50.	Flanagan TB, Sutton JA, Brown LM, Weinstein DA, Merlo LJ. Disordered Eating and Body Esteem Among Individuals with Glycogen Storage Disease. JIMD Rep. 2015;19:23-9.

51.	Hassall S, Smith DM, Rust S, Jones SA, Wittkowski A. "Why them, why me, why us?" The experiences of parents of children with lysosomal acid lipase deficiency: an interpretative phenomenological analysis study. Orphanet J Rare Dis. 2022;17(1):193.

52.	Cederbaum JA, LeMons C, Rosen M, Ahrens M, Vonachen S, Cederbaum SD. Psychosocial issues and coping strategies in families affected by urea cycle disorders. J Pediatr. 2001;138(1 Suppl):S72-80.
